# Editorial: Pharmacological and Immunological Action of Bacteriophages: Focus on Phage Therapy

**DOI:** 10.3389/fphar.2022.856542

**Published:** 2022-04-20

**Authors:** Mayank Gangwar, Subhash Karn, Sanjay Chhibber, Elizabeth Kutter, Gopal Nath

**Affiliations:** ^1^ Department of Microbiology, Institute of Medical Sciences, Banaras Hindu University, Varanasi, India; ^2^ Department of Microbiology, Basic Medical Sciences, Panjab University, Chandigarh, India; ^3^ The Evergreen State College, Olympia, WA, United States

**Keywords:** bacteriophages, phage therapy, antimicrobial resistance, phage immunology, pharmacology

Phage pharmacology can be studied under two major aspects *viz.* pharmacodynamics (i.e., a drug’s impact on the body) and pharmacokinetics (the body’s impact on a drug). Pharmacodynamics of phages mainly focuses on the phage titer used to kill the infection-causing bacterial cells; thus, a number of phages or phage cocktail standardization are very important before administration or initiation of phage therapy. In addition, the phage solution must be free from any type of pyrogenic substances that could cause an altered immune response. Pharmacologically, phages can be treated as very selective and specific toxic antibacterial agents (Carlton et al., 2005). These particular phages showed varied body’s humoral and innate immune responses that depend on the type of phage and the infection. On the other hand, phage pharmacokinetics mainly deals with the phage dosage or phage cocktail, which is needed to reach the target site, infect the pathogens, and finally for the bacterial eradication (Dąbrowska et al., 2018; Danis-Wlodarczyk et al., 2021).

Unlike drugs, bacteriophages are the bacteria viruses, not chemotherapeutics, and they are mainly employed in combating the bacterial infection, *i.e.,* phage therapy (Sulakvelidze et al., 2001). Overall, this editorial research topic, focuses on phage therapy and emphasizes the latest research concerning the potential therapeutic properties, especially pharmacology and immunology, against infectious multidrug-resistant microbes. Overall, an advanced immunological and pharmacological understanding of phage therapy should encourage and allow researchers to develop phages as antimicrobial “drugs” with improved design and therapy protocols. Phage therapy pharmacological concept is unique in contrast with antibiotics because phages are viruses that impact individual bacterial cells. In addition, bacteriophages can also amplify phage numbers *in situ*, making it an ideal future candidate against multi-drug resistant microbes with its unique and traditional immunological and pharmacological understanding.

Bacteriophages are widespread in the human body, possibly implying that the immune system might not perceive them as a threat (Manohar et al., 2019). Phages appear to adhere to the mucosal surfaces of a wide range of animals, reducing microbial colonization and pathology at these interfaces and providing a layer of immunity that is not acquired from the host (Barr et al., 2013). It has also been hypothesized that lytic phages help to maintain the microbiome’s variety and resilience by regulating its makeup (Krut and Bekeredjian-Ding, 2018). Researchers’ concerns mainly focused on phage therapy, which may alter the microbiome’s composition, or that the preexisting immune response to natural phages may interfere with phage therapy, which must be addressed in relevant models to clarify the *in vivo* pharmacological relevance. Thus, clear evidence regarding an altered immune response has yet to be established (Navarro and Muniesa, 2017).

Antigen-presenting cells (APCs) must process and present phages to T cells to trigger the generation of antibodies (Abs) and produce a long-lasting memory response. Several mechanistic theories have been proposed to explain how phages manage to get past epithelial barriers and into the generally sterile lymphoid organs: 1) phage particle transcytosis *via* epithelial cells, 2) epithelial barrier circumvention *via* a Trojan horse mechanism in which phages hide inside bacteria, 3) direct sampling of luminal material by intestinal dendritic cells, and 4) phage translocation across a damaged epithelial barrier (Duerkop and Hooper, 2013; To et al., 2017). The presence of phage-specific Abs can impair therapeutic efficacy, which is especially important in chronic infections because repeated treatments with the same phages boost the humoral immune response and can start the process of induction (Krut and Bekeredjian-Ding, 2018). Phage-mediated activation of innate immune cells is mainly based on recognizing phage-derived DNA and RNA by pattern recognition receptors (PRR). Therefore, specific PRR engagement and the extent of immune activation will differ depending on the phage type, dose, and nucleic acid synthesis activity.

The present research topic focused on phages, which has highlighted the current immunology and pharmacology of phage therapy along with its related advancements. This issue comprised a detailed case report, opinion, and a review article on bacteriophages.

Gondil and Chhibber, 2021 focused on the encapsulation system of bacteriophage and endolysin, which is found to be a promising strategy to improve therapeutic outcomes of phage therapy. Phage and endolysins’ encapsulation can improve various pharmacokinetic parameters with improved host immune response. The authors systematically discussed the role and advantages of endolysins (phage-borne lytic proteins) such as improved specificity, modular structure, rapid host lysis, and reduced chances of resistance. The authors mentioned various advanced encapsulation systems such as natural polymers, synthetic polymers, liposomes, nanosphere, and electrospun fibers. Thus, this type of encapsulation for phages protects from digestive enzymes and bile juices and provides permeability to the mucous lining. However, the endolysin delivery system of phages faced more challenges than phage delivery due to its proteinaceous nature and labile enzymatic activity. Thus, organic solvents play a major role in delivering the encapsulation of phages. The authors also mentioned the challenges and benefits of the endolysin delivery system; however, phage encapsulation system has been successfully reported in many clinical applications. As reported worldwide, these novel phages and endolysin delivery systems would be a promising method of drug delivery to combat multidrug-resistant infections.

Johri et al. investigated a case report of successful phage therapy against Chronic Bacterial Prostatitis (CBP) in a 33-year-old male. Before the start of the phage therapy, the patient underwent multiple courses of antibiotics treatment without any long-term clinical benefits. The case report stated the culture of prostatic secretion and semen samples with the presence of Staphylococcal species such as *Methicillin-resistant Staphylococcus aureus (MRSA)* and *Staphylococcus haemolyticus, Enterococcus faecalis, and Streptococcus mitis*. Thus, specific phage preparations were finalized from Eliava Institute and were administered as oral liquid, rectal suppositories, and urethral instillations. The case defined significantly decreased symptoms after the treatment period (approximately 2 months) such as high body temperature, weakness, night sweating, and chills. Thus, phage therapy can be proven to be a better alternative in CBP cases with improved clinical manifestations.

Kaur et al. systematically reported the role of nanotechnology in phage encapsulation, which can be an ideal pharmaceutical formulation approach in overcoming the pharmacological barriers such as less bioavailability, low stability, targeted delivery, inactivation of active phages, poor *in-vivo* retention, maintenance of viability, neutralization by the immune system, and poor penetration. Instead of focusing on nano-encapsulation, the authors mentioned a detailed insight about the recent nanotechnologies such as lipid-based nano-carriers, microfluidic, surface modification, nano-emulsions with integrated microfluidics for phage-cocktails, phage-loaded nanofibers using electrospinning method, and smart drug delivery platforms to control the high phage counts as required.

Thus, overall, in this alarming crisis due to multiple drug resistance microbes, the development of newer antibiotics alternatives with improved efficacy is the need of the hour. Phage therapy is one of the new rays of hope among the existing alternatives. The major limitations, such as immunological and pharmacological barriers, in front of phage therapy can be overcome by using these advanced formulation and drug targeting approaches. The immune system is one of the major challenges for scientists working on phage therapy, however lack of social acceptance due to minimal public awareness and unacceptable regulatory guidelines are the major hurdles to addressing and establishing phage therapy as one of the safest alternatives for MDR microbes and their associated infections. The aim of this research topic was to address the unique mechanism and pharmacological challenges in phage therapy. Finally, this topic offers an in-depth opinion and case report of phage therapy along with the successful delivery approaches of phages, which may inspire many researchers worldwide to continue working in or enter the science of phage preparation, formulations, and targeted delivery against various uncurable infections.
